# Machine learning-based prediction of well performance parameters for wellhead choke flow optimization

**DOI:** 10.1038/s41598-025-27851-8

**Published:** 2025-12-19

**Authors:** Ali Akbari, Fatemeh Ghazi, Yousef Kazemzadeh

**Affiliations:** Department of Petroleum Engineering, Faculty of Petroleum, Gas, and Petrochemical Engineering, Persian Gulf University, Bushehr, Iran

**Keywords:** Flow-rate prediction, Evolutionary optimization algorithms, Choke size, Liquid production rate, Machine learning, Energy science and technology, Engineering, Mathematics and computing

## Abstract

Accurate measurement and forecast of fluid flow rates in production wells are important to the estimation of hydrocarbon recovery, attainment of stable and controllable flow regimes, and optimization of production plans. Wellhead chokes, or pressure control valves, find widespread use in the hydrocarbon industry for two major reasons: provision of a stable downstream pressure and creation of the necessary backpressure for balancing gas well productivity and controlling in-well pressure drops. Over the past fifty years, numerous multiphase flow models and empirical correlations have been developed to estimate flow rates under a wide range of fluid properties, flow regimes, and pressure drop conditions. None of these models is deemed to be globally applicable to every region because each has inherent measurement errors that limit the accuracy of predictions for well performance parameters. In this study, three machine learning algorithms—Convolutional Neural Network (CNN), Multilayer Perceptron (MLP), and Radial Basis Function Network (RBFN)—were employed to predict well performance parameters. The dataset consisted of 182 samples for each of the five input parameters—liquid production rate (QL), wellhead pressure (Pwh), choke size (D64), basic sediment and water content (BS&W), and gas–liquid ratio (GLR)—resulting in a total of 910 data points. Among the tested models, MLP demonstrated the highest predictive performance, achieving R^2^ values of 0.9985 (training), 0.9856 (validation), and 0.9936 (testing). Four error metrics—Root Mean Square Error (RMSE), Mean Square Error (MSE), Mean Absolute Percentage Error (MAPE), and Mean Absolute Error (MAE)—were used for evaluation. For the MLP model, RMSE values of 0.0024 (training) and 0.0057 (testing) were obtained. The dataset was split into training and testing sets with a ratio of 70:30.

## Introduction

Permanent wellhead chokes are critically required equipment in most oil, gas, and condensate production wells and form the foundation for production control and optimization. They safely and stably control operating conditions by stabilizing and controlling single-phase flow or multiphase flow production through the application of sufficient backpressure on the reservoir without causing potential formation damage^1–3^.

Their worth extends beyond the mere regulation of production levels. Chokes directly contribute to the integrity and safety of surface facilities, controlling phenomena like water and gas coning, reducing sand and solid particle production, and permitting flexible control of production levels and ultimate resource recovery. In doing this, they enhance reservoir performance and life. Depending on reservoir conditions and the operational requirements, chokes are of two broad types: fixed (positive) chokes with a fixed internal diameter for constant long-term production, and adjustable (variable) chokes with the potential to change the orifice diameter, e.g., flow control valves^4–6^.

Flow through a wellhead choke will typically be in either critical or subcritical flow regimes, depending on fluid properties and downstream pressure conditions. Fluid velocity in the critical flow regime will be sonic velocity, and the flow rate will be independent of the downstream pressure. It is mainly true if the upstream pressure is at least 70% higher than the downstream pressure, or the downstream-to-upstream pressure ratio is less than or equal to 0.588. Subcritical flow rate depends on upstream and choke pressure difference, and variations in the upstream pressure will influence the downstream pressure^6,7^.

By prevailing in fines and sand, subcritical conditions set a boundary rate below which solids deposition is greatly increased. In sand-bearing or fines-bearing reservoirs, it is therefore better to be under subcritical conditions^8^.

Pressure drops across the choke and across the production string has also have the secondary effect of reducing the fluid to its bubble point and causing two-phase flow. The critical liquid production rate will be a function almost entirely of upstream pressure, gas–liquid ratio (GOR), and choke size, while in subcritical conditions the synergy of the latter two with the differential pressure controls the flow rate^9^.

It is required to fairly accurately predict the transition from subcritical to critical flow in order to reach the optimum choke design and operation. Downstream pressures are lowered to as low as 50% or even 5% of upstream pressure in some critical flow applications. It is one of the flagship challenges in this regard to predict two-phase flow rate from measurable parameters, i.e., GOR, bean size (choke size), pressure, and fluid properties^10^. There are two types of approaches to multiphase flow prediction by chokes such as analytical models or empirical correlations with strengths and weaknesses typical of it.

ML has become a unifying tool in petroleum engineering, where its adaptability allows it to address diverse challenges while maintaining direct relevance to wellhead choke flow optimization. For instance, hydraulic fracturing evaluation using ML not only improves fracture geometry prediction but also provides crucial input parameters such as post-fracture productivity and reservoir flow behavior, both of which influence choke flow performance and well deliverability^11^. Similarly, the calculation of hydrogen dispersion in cushion gases with ML and the modeling of dispersion coefficients in porous media for hydrogen storage generate insights into multiphase and multicomponent flow mechanisms that are directly analogous to the complex fluid interactions occurring across wellhead chokes^12^. Furthermore, sustainable water management in hydraulic fracturing creates higher-quality production data and minimizes formation damage, leading to more stable flow conditions that can be better predicted and optimized using choke flow models^13^. Even the application of RFID technology in petroleum engineering, by enabling real-time data collection and monitoring, can strengthen machine learning frameworks used for choke optimization through more accurate and timely operational datasets^14^.

Beyond these operational analogies, several ML applications provide direct methodological parallels that reinforce choke flow optimization studies. For example, estimating minimum miscible pressure (MMP) in CO_2_ injection using ML reflects the same requirement for precise prediction of pressure-dependent parameters, which is central to choke performance modeling^15^. Likewise, using ultrasonic and microwave methods to mitigate wax deposition addresses flow assurance problems that affect wellhead pressure drops and, when coupled with ML prediction, can enhance choke setting strategies for uninterrupted flow^16,17^. Finally, enhanced water saturation estimation using ML provides more accurate reservoir characterization, thereby improving input parameters such as fluid saturation, relative permeability, and flow potential, all of which are critical to predicting choke-controlled production rates^18^. Collectively, these diverse ML-driven applications demonstrate that whether addressing subsurface properties, surface facilities, or flow assurance, machine learning consistently contributes to improving the accuracy, robustness, and real-time adaptability of well performance prediction for choke flow optimization.

Recent studies in fluid mechanics have highlighted the strong influence of flow geometry and discharge variations on fluid behavior. In 2020, Azma and Zhang^19,20^ demonstrated through CFD simulations that changes in discharge ratio and tributary width significantly alter turbulence, recirculation zones, and velocity distribution in channel confluences. These findings are analogous to wellhead choke systems, where variations in choke diameter and gas–liquid ratio directly affect flow regime transitions and stability. Such studies underscore the importance of geometric and dimensionless parameters, such as Froude number, in predicting multiphase flow performance.

In parallel, machine learning and hybrid AI methods have gained prominence as efficient alternatives to computationally intensive CFD approaches. In 2023, Azma et al.,^21^ introduced a fuzzy-based bee algorithm that accurately reproduced CFD-driven nanofluid data with reduced computation time, while in 2025, Azma et al.,^22^ applied hybrid SVR-PSO and SVR-GA models to predict hydraulic discharge coefficients with superior accuracy compared to traditional methods. These works align closely with the present study, where GA-optimized ANN models were employed for choke flow prediction. Collectively, they demonstrate that data-driven models not only capture nonlinear flow dynamics effectively but also provide scalable and reliable solutions for optimizing complex engineering systems.

The novelty of this study lies in the integrated application of three widely used ANN architectures—MLP, RBFN, and CNN—for predicting liquid production rate (QL), with their hyperparameters optimally tuned using a Genetic Algorithm (GA). Moreover, the model inputs, comprising wellhead pressure (Pwh), choke size (D64), basic sediment and water content (BS&W), and gas–liquid ratio (GLR), were selected based on their operational significance. A comprehensive dataset containing 910 records was employed, and model evaluation was conducted using a combination of error metrics (MSE, RMSE, MAE, and MAPE) and advanced visualization techniques (KDE, learning curves, regression plots, error distribution, and SHAP (SHapley Additive exPlanations) analysis). This multifaceted approach ensures both high accuracy and interpretability, offering a novel and robust solution for data-driven forecasting in petroleum production systems.

## Methodology

### Data collection and processing

In this study, the dataset was extracted from the research conducted by Ghorbani et al.^9^, which focused on predicting liquid flow-rate performance through wellhead chokes using genetic and solver optimization algorithms. The dataset comprises 182 wellhead test records obtained from seven production wells, offshore southwest of Iran. These wells penetrate three distinct reservoir zones with varying fluid properties. In the field measurements reported by Ghorbani et al., production tests were carried out using standard wellhead choke assemblies. The well stream flowed through the choke, where the effective bean size was adjusted and recorded. Pressure gauges were installed at the wellhead upstream of the choke to measure flowing wellhead pressure (Pwh). The multiphase stream was then directed to a test separator, where liquid and gas flow rates were measured separately with calibrated flow meters. The gas–liquid ratio (GLR) was subsequently determined from these measurements. Representative liquid samples were collected from the separator for laboratory analysis to determine basic sediment and water (BS&W) content using centrifuge and ASTM-based procedures. This workflow ensured that all key input parameters—Pwh, D64, QL, GLR, and BS&W—were obtained with standard industry practices and acceptable accuracy.

Each test record includes five key parameters: liquid production rate (QL), wellhead pressure (Pwh), choke size (D64), basic sediment and water content (BS&W), and gas–liquid ratio (GLR). Since each parameter contains 182 data points, the total number of individual data entries used in this study is 910.

To assess the distribution characteristics and overall quality of the input dataset, the results of this analysis are summarized in Table 1, which presents key descriptive statistics for each input parameter, including the maximum, minimum, range, median, first quartile (Q1), third quartile (Q3), mean, variance, skewness, and kurtosis. These statistical indicators provide valuable insights into the central tendency, variability, and symmetry of the data, thereby facilitating a better understanding of the dataset’s structure and its suitability for further modeling and analysis.

**Table 1:** Data statistics.

	QL	Choke	Pwh	BS&W	GLR
Max	34,450	64	881	66	885
Min	205	25.6	133	0.02	36
Range	34,245	38.4	748	65.98	849
Median	8400	64	508	18.3	227
Q1	4692	51.2	442.5	9.75	119
Q3	13,710	64	581	28.50	358.75
Mean	9734.28	57.67	521.33	19.60	251.67
Variance	47,888,399	117.8698	14221.02	173.39	25403.88
Skewness	1.12	-1.63	0.49	0.40	1.01
Kurtosis	1.44	1.29	0.75	-0.02	1.42
Standard deviation	6920.14	10.86	119.25	13.17	159.39

While the present study employed five key operational parameters (QL, Pwh, D64, BS&W, and GLR) as model inputs, other dynamic variables such as temperature, emulsion content, and API gravity were not included due to limitations in the available dataset. These parameters are known to influence multiphase flow behavior and fluid properties, and their exclusion may restrict the model’s predictive accuracy under varying reservoir conditions. Nevertheless, the current parameter set captures the most frequently monitored variables at the wellhead and provides a practical foundation for operational deployment. Future research will expand the feature set by incorporating additional dynamic variables through broader field data collection, which is expected to further improve model robustness and generalizability across different operating environments.

**Fig. 1:**
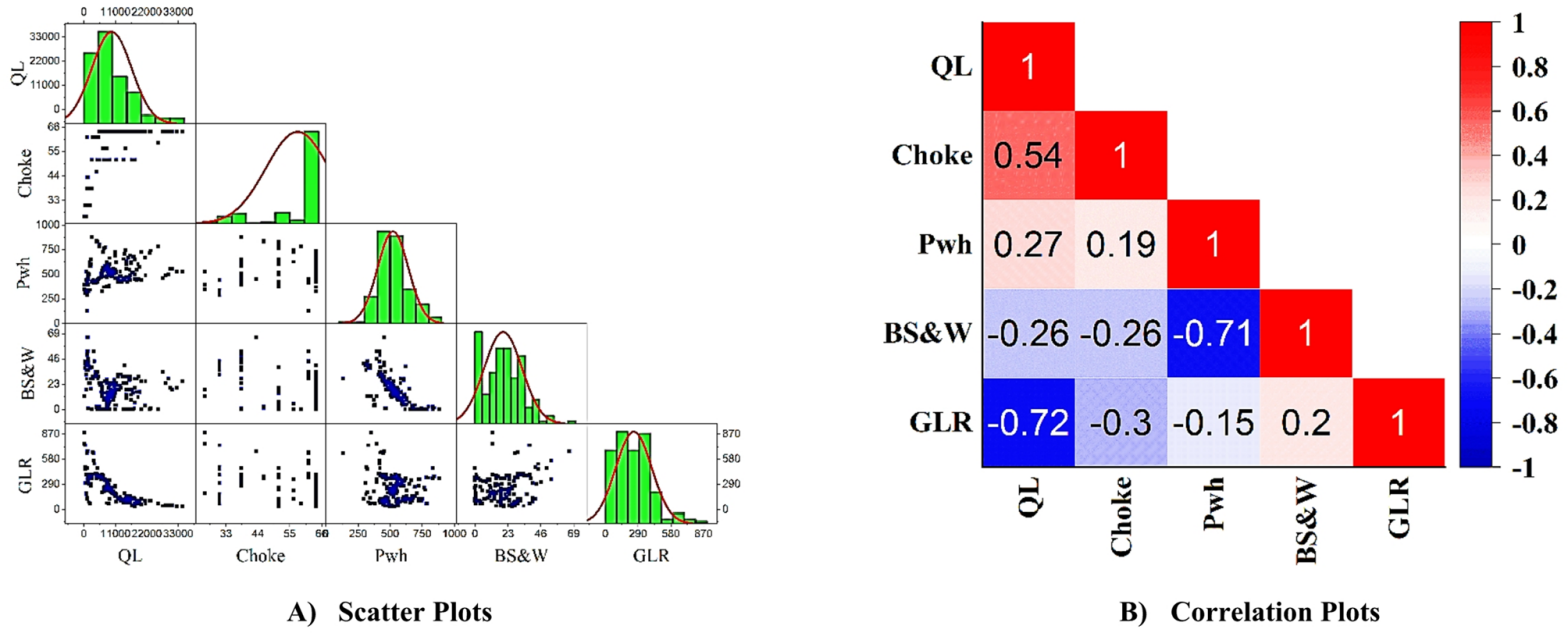
Scatter plots and correlation plots.

To enhance the analysis of the data, Correlation Plots and Scatter Plots were generated (Fig. 1). The correlation plot allows for the examination of linear relationships between input variables and helps identify the strength and direction of associations among different parameters. Meanwhile, the scatter plot provides a visual representation of the data distribution and potential patterns between two variables. These plots not only improve understanding of the structure and interactions among variables but also assist in detecting outliers, unusual trends, and possible nonlinear relationships. Such visual analyses play a crucial role in preparing the data for modeling and improving the accuracy of statistical predictions.

Figure 1B presents the correlation matrix of the input and output parameters. As shown, QL exhibits a strong negative correlation with GLR (− 0.72), indicating that higher gas–liquid ratios reduce liquid production rate. A moderate positive correlation is observed between QL and choke size (0.54), confirming the role of choke geometry in enhancing liquid flow. In addition, BS&W shows a significant negative correlation with Pwh (− 0.71), suggesting that higher water and sediment content may lower wellhead pressure. Other parameters, such as QL–Pwh (0.27) and Choke–Pwh (0.19), display weaker correlations. These results highlight the dominant influence of choke size, GLR, and BS&W on choke flow behavior.

Normalization was a critical preprocessing step applied across all three neural network architectures used in this study—RBFN, MLP, and CNN—to enhance training stability and convergence. Min–Max normalization was used to scale the input features uniformly within the [0, 1] range, thereby preventing variables with larger numerical scales from dominating the learning process. To ensure model generalization and avoid data leakage, normalization parameters were calculated exclusively from the training set and then applied to the validation and test sets. After prediction, the normalized outputs were transformed back to their original scale using the inverse Min-Max formula, allowing for direct comparison between predicted and actual target values in their original units. Overall, the consistent application of Min–Max normalization contributed to faster convergence, improved numerical stability, and a standardized input space for fair model evaluation.

### Artificial neural network

#### CNN

Convolutional Neural Networks (CNNs) are deep networks that mimic the human visual system and can learn and detect dominant features independently from structured data such as images, voice, and time-series data. In parallel with the natural grid-like nature of the aforementioned data, CNNs learn hierarchically at the same time the features. Their inherent capacity for small input translation, scaling, distortion, or noise has rendered them of hitherto unforeseen application in image processing, computer vision, and signal analysis^23^. There are three forms of CNN architecture in use:

- Convolutional layers, to extract features,
- Pooling layers, to reduce data and reduce dimensions,
- Fully connected layers, for prediction or classification.

There are numerous learnable small-sized filters relative to the input in a complex layer, which filter data sequentially. The dot product of the corresponding input segment and the filter weights is computed at every spatial location to produce feature maps. It allows the network to learn local features such as edges, texture, and higher-order form. As opposed to the traditional neural networks where a neuron is connected with a scalar weight, in CNNs every neuron is given a two-dimensional size-k kernel weight matrix.

Subsequent to the feature extraction, pooling layers are typically used to reduce data size and highlight the important features. The layers sum up or downsample from local nearby regions and reduce output size without losing detail. Taking the maximum of a region being max pooling is among the most widely used strategies for reducing computational cost as well as improving tolerance for small change in input^24,25^.

Lastly, the fully connected layers will also transfer what is achieved through learning the features to the output, i.e., class labels or numerical prediction through nonlinear activation functions:

1 $$\:y=f(Wx+b)$$

where $\:x$ is the input vector, $\:W$ is the weight matrix, $\:b$ is the bias, and $\:f$ is the activation function. All the neurons in all these layers are feedforward to the neurons in the previous layer.

For 1D inputs, such as sensor measurements or time-series, 1D CNNs are used. They are minimal and lightweight substitutes to 2D CNNs, with enormously fewer parameters, resulting in faster training as well as inference. Simple 1D CNN models consist of a single or two convolutional layers, and hence they are highly suitable for real-time and cost-constrained applications. The forward pass of a 1D convolutional layer may be written as:

2 $$\:{X}_{k}^{l}={b}_{k}^{l}+\sum\:_{i=1}^{{N}_{i-1}}conv1D({W}_{ik}^{l},{S}_{i}^{l-1})$$

3 $$\:{Y}_{k}^{l}=f\left({X}_{k}^{l}\right)$$

conv1D is the convolution function with zero-paddling disabled (Invalid) and $\:{Y}_{k}^{l}$ is the output of activation^26,27^.

CNNs are generally trained with the backpropagation algorithm. Error in the output layer is initially estimated with a loss function like mean squared error (MSE):

4 $$\:{E}_{MSE}=\frac{1}{{N}_{L}}\sum\:_{i=1}^{{N}_{L}}{({t}_{ip}-{y}_{i}^{L})}^{2}$$

The error is thus back-propagated through the network across the network through the chain rule for computing the gradient of loss with respect to weights. Iteratively, the weights are modified so that the error can be minimized. In this way, CNNs can automatically learn weights in such a way that feature detection and classification can be accomplished in both stable and accurate ways^28–30^.

#### MLP

Multilayer Perceptron (MLP) is probably the most commonly used artificial neural network structure, roughly defined as having nonlinear mappings between highly complex input variables to output variables. Structural configurations of an MLP include a sequence of layers, an input layer, certain hidden layers, and an output layer. All the neurons in a hidden layer receive synaptic inputs from the next and previous layer neurons, and each of them is given weight. All the neurons except the input layer also receive a bias term. All the weights and biases are updated constantly during training to minimize the network’s prediction error.

One idiosyncrasy of MLPs is implementing the use of nonlinear activation functions where the network is able to learn nonlinear patterns and relations between data that can’t be linearly represented. Common activation functions include sigmoid, tanh, and rectified linear unit (ReLU). The output layer for regression problems will use linear activation as a try to create continuous-valued outputs^31^.

The MLP training generally has two general steps:

Feedforward propagation Input data is forwarded from input layer to the output layer via the hidden layers. Every neuron performs a weighted summation of inputs, adds its own bias and places the given activation function in order to produce its output.

Backpropagation The difference between desired and target output is used to find the difference, and the error gradient with the biases and the weights is found. Basic gradient descent or more complex algorithms such as Levenberg–Marquardt (LM), Bayesian Regularization (BR), or Scaled Conjugate Gradient (SCG) is used to modify the biases and the weights^32^.

Mathematically, an MLP with two hidden layers first hidden layer with hyperbolic tangent (tansig) activation function, second hidden layer with logistic sigmoid (logsig) activation function, and output layer with linear activation (purlin) can be expressed symbolically as:

5 $$\:Tansig=tanh\::h\left(x\right)=\frac{{e}^{x}-{e}^{-x}}{{e}^{x}+{e}^{-x}}=\frac{2}{1+{e}^{-2x}}-1$$

6 $$\:linear=pureline=h\left(x\right)=x$$

7 $$\:sigmoid=logsig\::h\left(x\right)=\frac{{e}^{x}}{{e}^{x}+1}$$

Consider an MLP with two hidden layers and logsig and tansig activation functions for the two hidden layers and purlin for the output layer, respectively. The output of the model can be calculated by the following formula:

8 $$\:output=purlin({w}_{3}\times\:\left(logsig\left({w}_{2}\times\:\left(tansig\left({w}_{1}\times\:x\right)+{b}_{1}\right)\right)+{b}_{2}\right)+{b}_{3}$$

The most important strengths of MLPs are that they can identify nonlinear relationships, have excellent generalization capability, and are universal to handle a very wide range of problems, such as time series forecasting, pattern recognition, natural language translation, speech understanding, and data modeling in high dimensions^10,33,34^.

#### RBFN

Radial Basis Function Neural Networks (RBFNNs) enable the construction of learning models that can be refined and optimized over time to solve the phenomenon at hand. RBFNNs utilize the radial basis functions as the main activation function in place of the conventional approach through the sigmoid activation functions^35^.

There are three levels in all RBFNNs: an input level, which receives raw input; a hidden level, composed of RBF nodes, each computing a nonlinear radial function of the inputs; and an output level, which generates the prediction. The networks are content to have the property of fast learning, minimal structural complexity, easy parameter tuning, and maximal learning capacity for approximating high-order input–output relations^36–38^.

For input $\:x\in\:{\mathbb{R}}^{d}$( in this d = 2), an RBF neural network with one output unit can be formulated as:

9 $$\:N\left( x \right) = \sum {_{{j = 1}}^{m} } \omega _{j} \phi \left( {\frac{{\left\| {x - c_{j} } \right\|^{2} }}{{\sigma _{j} }}} \right)$$

m = number of RBFs (or hidden nodes), $\:\left\| . \right\|$=denotes the Euclidean norm, $\:{\omega\:}_{j}$= connection weight from the $\:j-th$ hidden unit to the output unit, $\:{c}_{j}$= the prototype or center of the $\:j-th$, $\:\phi\:$= shape parameter of the $\:j-th$.

All the weights to the output neurons are summed to give $\:\theta\:={\left\{{\omega\:}_{j},{c}_{j},\phi\:\right\}}^{2}$total. The output biases are accommodated by a separate fixed-activation hidden neuron. Local activation is an intrinsic characteristic of RBFNNs—hidden nodes are most active when the input is nearest to their own centers. It gives excellent continuous function approximations without coordinate transformation or interpolation.

It is obtained by iteratively updating weights, centers, and radii of radial functions through empirical algorithms. The algorithm is highly adaptive and highly fast convergent, and RBFNNs are highly powerful to process highly complex in nature data sets^35,39,40^.

## Results and discussion

### Data division into training and testing sets

In this phase of the study, the raw petrophysical data were first collected and subjected to initial preprocessing steps. To investigate the impact of different data partitioning strategies on model performance, the dataset was systematically split into test sets ranging from 10% to 90% in increments of 10%. For each configuration, a designated portion served as the test set, while the remaining data were allocated for training and validation, with typically 10% of the remainder reserved for validation. This iterative approach allowed for consistent and comparable evaluations across all experiments.

Table 2 details the percentage and actual number of data points assigned to the training, validation, and test sets in each scenario. As described, the process began with a 10% test set and 90% for training and validation. The model was trained, and the root mean square error (RMSE) was calculated. This procedure was repeated, increasing the test set size by 10% at each step (i.e., 20%, 30%, …, up to 90%), and RMSE was recalculated accordingly. The primary objective was to determine the most effective data split ratio to optimize model accuracy and reliability.

**Table 2:** Detailed distribution of data samples across training, validation, and testing sets for each split scenario.

Test	No. of test sample	Train + validation	No. of training & validation samples
10%	91	90%	819
20%	182	80%	728
30%	273	70%	637
40%	364	60%	546
50%	455	50%	455
60%	546	40%	364
70%	637	30%	273
80%	728	20%	182
90%	819	10%	91

**Fig. 2:**
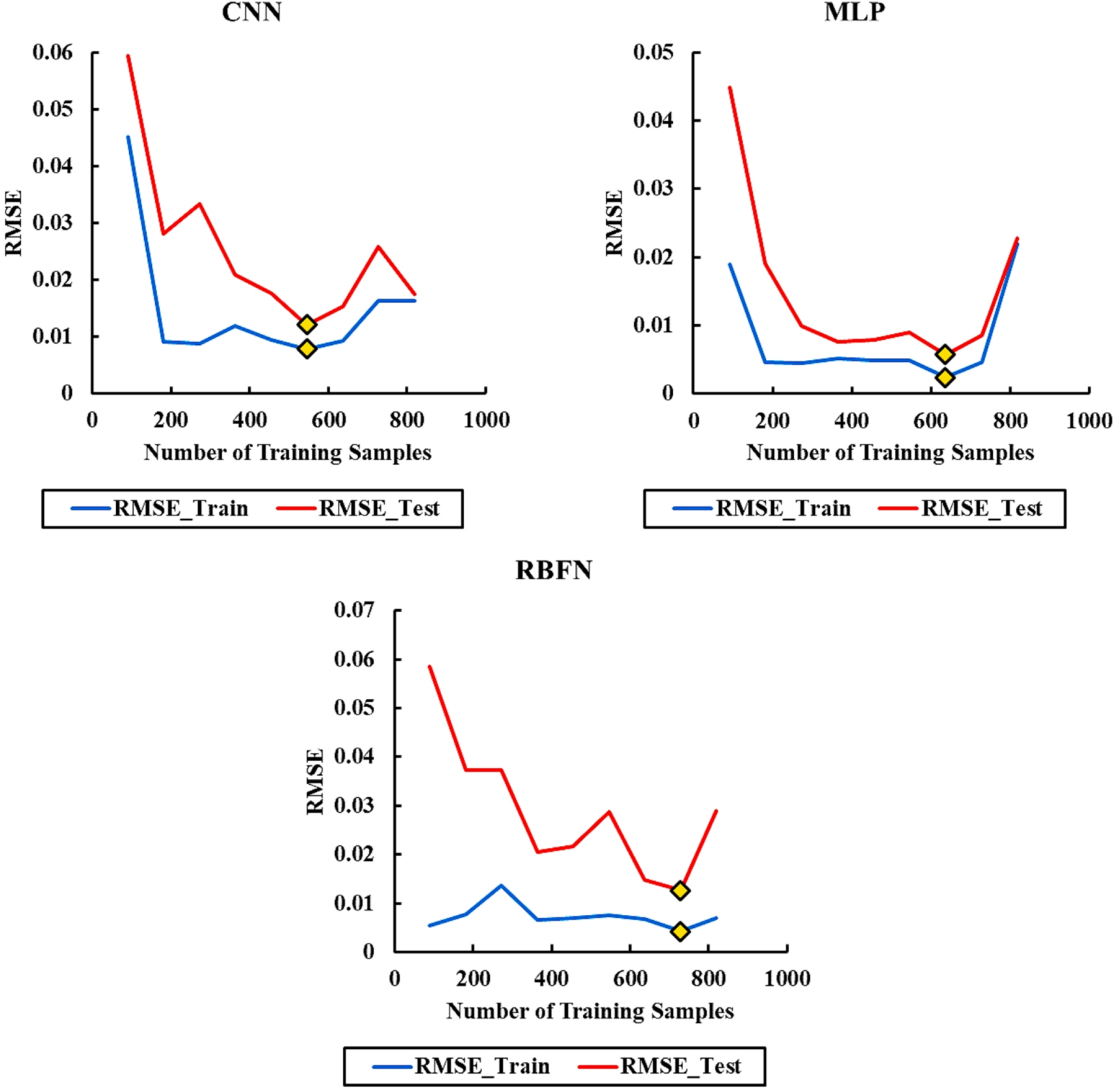
Learning curves illustrating RMSE as a function of training sample size for the RBFN, MLP, and CNN models. The red lines represent testing errors, while the blue lines indicate training errors. The trend highlights that increasing training data generally reduces prediction error, with MLP maintaining the lowest error across scenarios.

To evaluate the performance of each data split configuration, the Root Mean Square Error (RMSE) was used as the primary evaluation metric. Figure 2 presents the RMSE results for all tested data partitioning scenarios. Configurations that yielded the lowest RMSE values were considered the most effective, as they correspond to the smallest prediction errors. The scenarios with the minimum RMSE are highlighted with yellow markers, indicating the data split ratios that resulted in optimal model performance. All evaluations and computations in this study were performed using Python, leveraging its powerful libraries for data processing, model training, optimization, and visualization.

For the CNN algorithm, a total of 546 data points were selected for training, with the remainder used for testing. Similarly, for the MLP algorithm, 637 data points were used for training, and for the RBFN algorithm, 728 data points were allocated for training.

**Fig. 3:**
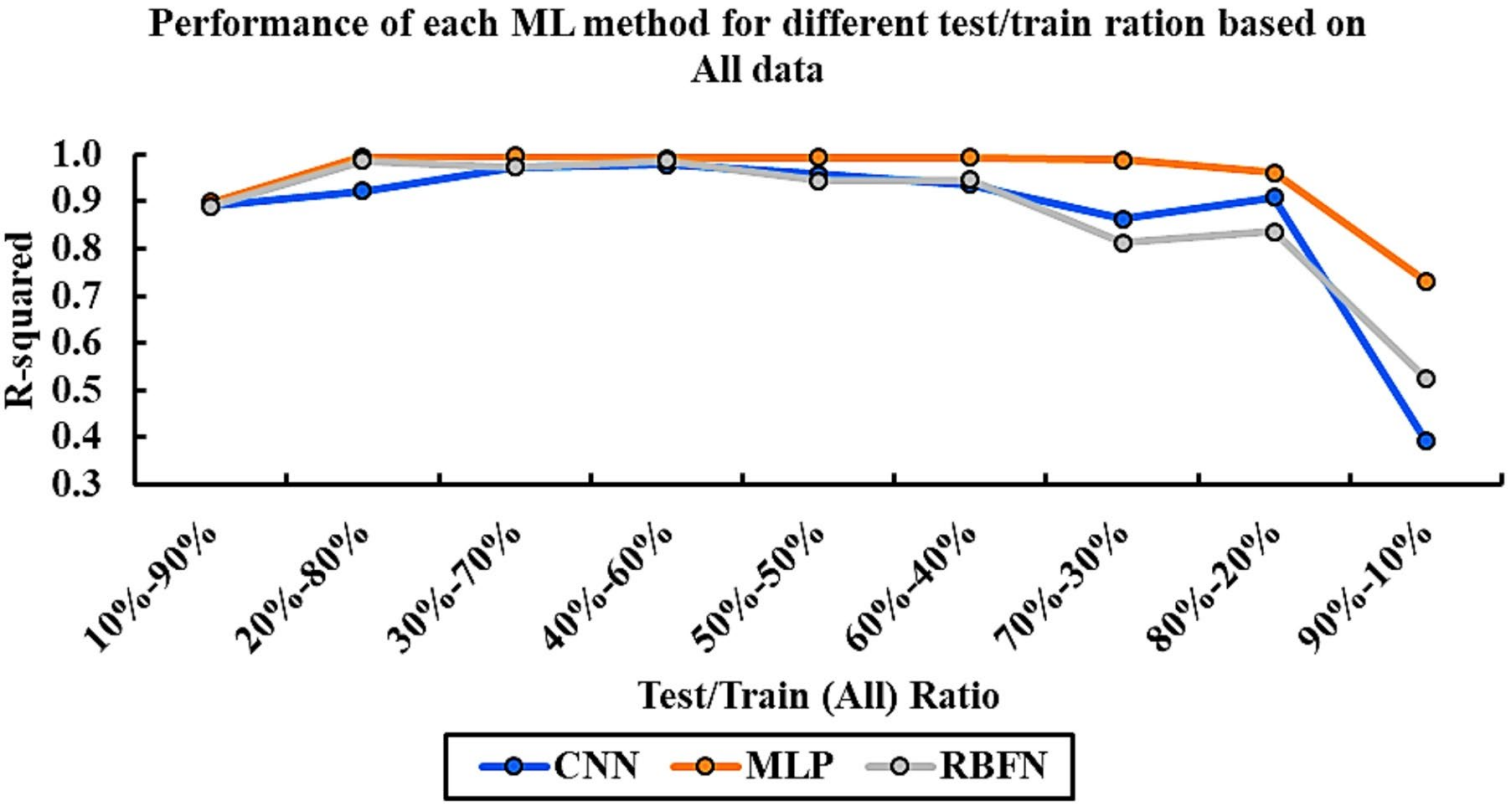
Comparative assessment of R^2^ values for CNN, MLP, and RBFN models under different training-to-testing data split ratios (10–90%). The results show that MLP achieves the most consistent accuracy across splits, CNN performs best at a 40–60 split, and RBFN requires larger training sets (80%) to reach acceptable performance.

To comprehensively and precisely assess the performance of the models, it is essential to evaluate all algorithms under consistent conditions. Figure 3 displays the R^2^ values for each algorithm across various data splitting scenarios between training and testing sets. This figure demonstrates the performance variations of each model as the training-to-testing data ratio varies from 10% to 90%. This method enables a straightforward comparison of model effectiveness, allowing identification of algorithms that exhibit greater accuracy and robustness under different data partition schemes. The R^2^ metric, being a vital indicator of prediction accuracy, plays a key role in this assessment and is used as the main criterion for selecting the most appropriate model for the given task.

Figure 3 summarizes the impact of different training-to-testing data split ratios on model performance. The results show that the CNN model achieved its highest accuracy when 40% of the data was allocated to testing and 60% to training and validation. In comparison, the MLP model performed best with a 30–70 split, highlighting its ability to generalize effectively with a slightly larger training set. The RBFN model, however, reached its optimal performance at a 20–80 split, indicating that it requires more training data to achieve reliable results. These findings emphasize that while each algorithm responds differently to the data partitioning strategy, the MLP consistently delivers the most robust performance across various scenarios, followed by CNN, with RBFN showing relatively weaker adaptability.

### Genetic algorithm

The Genetic Algorithm (GA) is widely recognized as an effective optimization tool for enhancing the performance of various machine learning models. By systematically exploring complex hyperparameter spaces, GA facilitates the identification of optimal model configurations, leading to improved accuracy and robustness across diverse datasets. This capability is particularly valuable in petroleum engineering, where the nonlinear nature of subsurface properties requires adaptable and precise modeling approaches. The optimized hyperparameters for each of the three algorithms examined in this study are detailed as follows:

#### Radial basis function network

In this study, the GA was utilized to optimize key hyperparameters of the RBFN model, namely the gamma value and the number of centers within the network. The gamma parameter was allowed to vary continuously between 0.01 and 10, enabling precise adjustment of the radial basis function spread. Meanwhile, the number of centers was limited to discrete values between 10 and 100. The optimization process spanned 200 generations, with a population of 40 potential solutions. In each iteration, five individuals were chosen as parents through a steady-state selection approach, which ensured that only a portion of the population was updated, preserving high-performing candidates. To enhance population diversity, a single-point crossover and random mutation were applied, with mutations affecting 50% of the genes. The evaluation of each candidate relied on minimizing the prediction error using a validation dataset. As GA inherently seeks to maximize fitness, the negative value of the error function was used as the objective. This method enabled the identification of hyperparameter configurations that significantly enhanced the accuracy and generalizability of the RBFN model.

#### Multilayer perceptron

For the MLP-based artificial neural network, GA was employed to fine-tune two primary hyperparameters: the learning rate and the number of neurons in the hidden layer. The goal was to minimize the Root Mean Square Error (RMSE) on the validation set. The learning rate was restricted to the interval 0.0001-0.1, and the hidden layer size was allowed to vary between 10 and 100 neurons. A custom objective function was developed to assign a high penalty to invalid parameter sets, ensuring feasible model configurations. The optimization process began with a population of 10 individuals and continued for 20 generations. Tournament selection with a pool size of three was used to ensure competitive pressure during evolution. New offspring were generated using a blended crossover technique with an alpha value of 0.5. Mutations, applied with a 30% probability, introduced Gaussian noise to both the learning rate and hidden layer size while maintaining the values within the defined boundaries. Ultimately, the configuration with the lowest validation error was selected as the optimal setup for the MLP model.

#### Convolutional neural network

In this case, GA was leveraged to optimize six crucial hyperparameters of the CNN model: learning rate, number of convolutional filters, kernel size, dropout rate, and the number of units in two fully connected layers. Each parameter was assigned a specific search space with defined step sizes—learning rate ranged from 0.00005 to 0.01 (step: 0.00005), number of filters from 16 to 128 (step: 8), kernel size between 2 and 5, dropout rate from 0.0 to 0.5 (step: 0.05), and dense layer units ranging from 64 to 256 and 32 to 128 (steps of 32 and 16, respectively). The genetic optimization proceeded over 100 generations with a population of 20 individuals. In each generation, eight parents were chosen through a steady-state selection method, allowing elite solutions to be retained and ensuring consistent convergence. The algorithm employed single-point crossover and random mutation (applied to 30% of the genes) to maintain genetic diversity and prevent premature convergence. The fitness function aimed to reduce the prediction error on the validation set, and an automatic stopping condition was implemented if zero error was achieved. This rigorous optimization scheme enabled precise tuning of the CNN’s architecture, significantly enhancing its performance through a thorough exploration of the parameter space.

Several key hyperparameters of the CNN, MLP, and RBFN models were optimized using a GA, each contributing significantly to model training and predictive performance. In the CNN, the learning rate controls the speed at which the model updates its weights, influencing both convergence speed and training stability. The number of filters determines how many distinct features can be detected in each convolutional layer, while the kernel size defines the spatial dimensions of these filters, affecting the granularity of local feature extraction. In the MLP, the learning rate again governs the speed of parameter updates, and the hidden layer size specifies the number of neurons in the hidden layers, directly impacting the model’s capacity to capture complex data relationships. For the RBFN, the gamma parameter defines the scope of influence of each radial unit, which affects the smoothness and flexibility of the model, and the number of centers determines how well the input space is represented by the radial basis functions. The final values of these hyperparameters, tuned through the genetic algorithm, are presented in Table 3, which shows the optimized hyperparameters for the artificial neural network models.

**Table 3:** Optimized hyperparameters for artificial neural network models using GA.

CNN	LearningRate	0.002
	Filters	48
	KernelSize	3
MLP	LearningRate	0.1
	HiddenLayerSize	67
RBFN	Gamma	0.7917
	Centers	99

To ensure reproducibility, the full architectural details of the CNN and MLP models are provided. The CNN model consisted of two 1D convolutional layers, the first with 48 filters and a kernel size of 3, followed by a second convolutional layer with 32 filters and a kernel size of 3. Each convolutional layer employed the ReLU activation function, and max pooling with a pool size of 2 was applied after the first convolutional block. The output of the convolutional layers was flattened and passed through two fully connected (dense) layers with 128 and 64 neurons, respectively, both using ReLU activation. A dropout rate of 0.3 was applied after each dense layer to mitigate overfitting. The final output layer contained a single neuron with linear activation for regression. The Adam optimizer with an initial learning rate of 0.002 was used for training, and MSE served as the loss function.

The MLP model was structured with an input layer of five neurons (corresponding to the five input parameters), followed by two hidden layers. The first hidden layer contained 67 neurons with the hyperbolic tangent (tansig) activation function, while the second hidden layer contained 45 neurons with a logistic sigmoid (logsig) activation function. A dropout rate of 0.2 was applied to each hidden layer. The output layer consisted of a single neuron with linear activation (purlin) to predict the liquid production rate (QL). The model was trained using the Adam optimizer with a learning rate of 0.1, and MSE was used as the loss function.

These architectural specifications, together with the GA-based hyperparameter optimization described earlier, ensure that the developed models are fully reproducible and can be reliably benchmarked in future studies.

The GA used for hyperparameter optimization was evaluated in terms of runtime and convergence behavior. On average, the GA required approximately 18–22 min to converge for the CNN model and 12–15 min for the MLP model when executed on a standard workstation (Intel Core i7 CPU, 12 GB RAM). The convergence curves indicated that the fitness function stabilized after 35–40 generations, confirming the efficiency of the search process. Although GA is computationally more intensive compared to simpler techniques, it provides broader exploration of the hyperparameter space and avoids premature convergence to local optima. For comparison, a grid search with the same parameter ranges required significantly longer computation time (up to 40 min) due to exhaustive evaluations, while Bayesian optimization achieved faster runtimes (~ 10 min) but occasionally converged to suboptimal solutions. These results demonstrate that GA offers a practical balance between accuracy and runtime, justifying its selection in this study for robust model optimization.

**Fig. 4:**
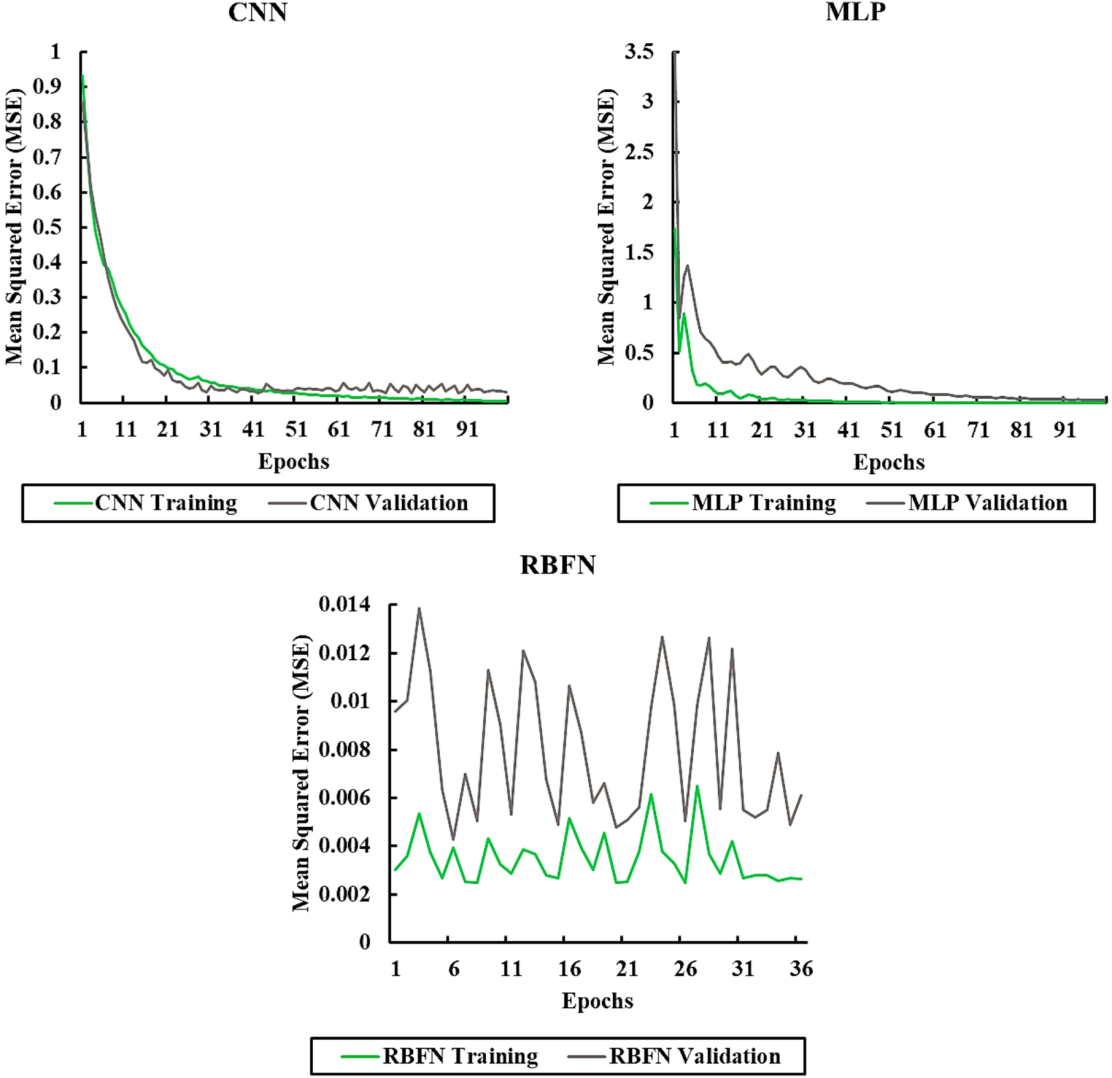
Learning curves showing training and validation MSE across epochs for CNN, MLP, and RBFN models. The MLP achieves the closest alignment between training and validation errors, indicating strong generalization, while RBFN converges quickly but at higher error levels.

Figure 4 shows the learning curves of the CNN, MLP, and RBFN models, plotting the mean squared error (MSE) against the number of training epochs for both training and validation sets. The CNN model demonstrates relatively smooth convergence, though its validation error remains higher than that of the MLP, indicating a moderate gap between training and testing performance. The MLP model achieves the lowest validation error overall, with its training and validation curves closely aligned, confirming strong generalization capability and minimal overfitting. In contrast, the RBFN model converges more quickly but stabilizes at a higher validation error compared to MLP and CNN, reflecting its lower predictive accuracy. These patterns highlight the superior balance of learning efficiency and robustness in the MLP model, while CNN provides competitive but slightly less stable performance, and RBFN exhibits faster but less accurate convergence. The application of early stopping further ensured that all models were trained up to their optimal point, preventing unnecessary computations and reducing the risk of overfitting.

Table 4 reports the error values recorded at each epoch for both the test and validation datasets. This data provides a clear view of how the models’ performance changed throughout the training process, highlighting trends in error decrease or escalation across different stages of learning.

**Table 4:** Epoch-wise MSE values for the test and validation datasets of each model.

Model	Epoch	Training (MSE)	Validation (MSE)
CNN	100	0.0046	0.0298
MLP	100	0.0012	0.0309
RBFN	36	0.0026	0.0061

In addition to predictive accuracy, the operational feasibility of machine learning models is strongly influenced by inference time, latency, and compatibility with existing supervisory systems. In the present study, the average inference time of the trained MLP and CNN models was approximately 25–40 ms per sample on a standard CPU, which is sufficiently fast for near real-time decision-making at the wellhead. Such low latency ensures that predictions can be seamlessly integrated into supervisory control and data acquisition (SCADA) platforms or deployed on edge devices without requiring high-performance computing infrastructure. Moreover, the relatively lightweight structure of the optimized models makes them suitable for deployment in digital oilfield frameworks where rapid choke adjustment is essential. While full-scale integration with SCADA and field automation systems was beyond the scope of this study, the reported inference efficiency demonstrates that the proposed models are industrially viable and can be incorporated into production monitoring and optimization workflows. Future research will extend this work by testing the models under real-time SCADA environments and validating their robustness under different operating conditions.

### Performance of each method in the training and testing phases

**Fig. 5:**
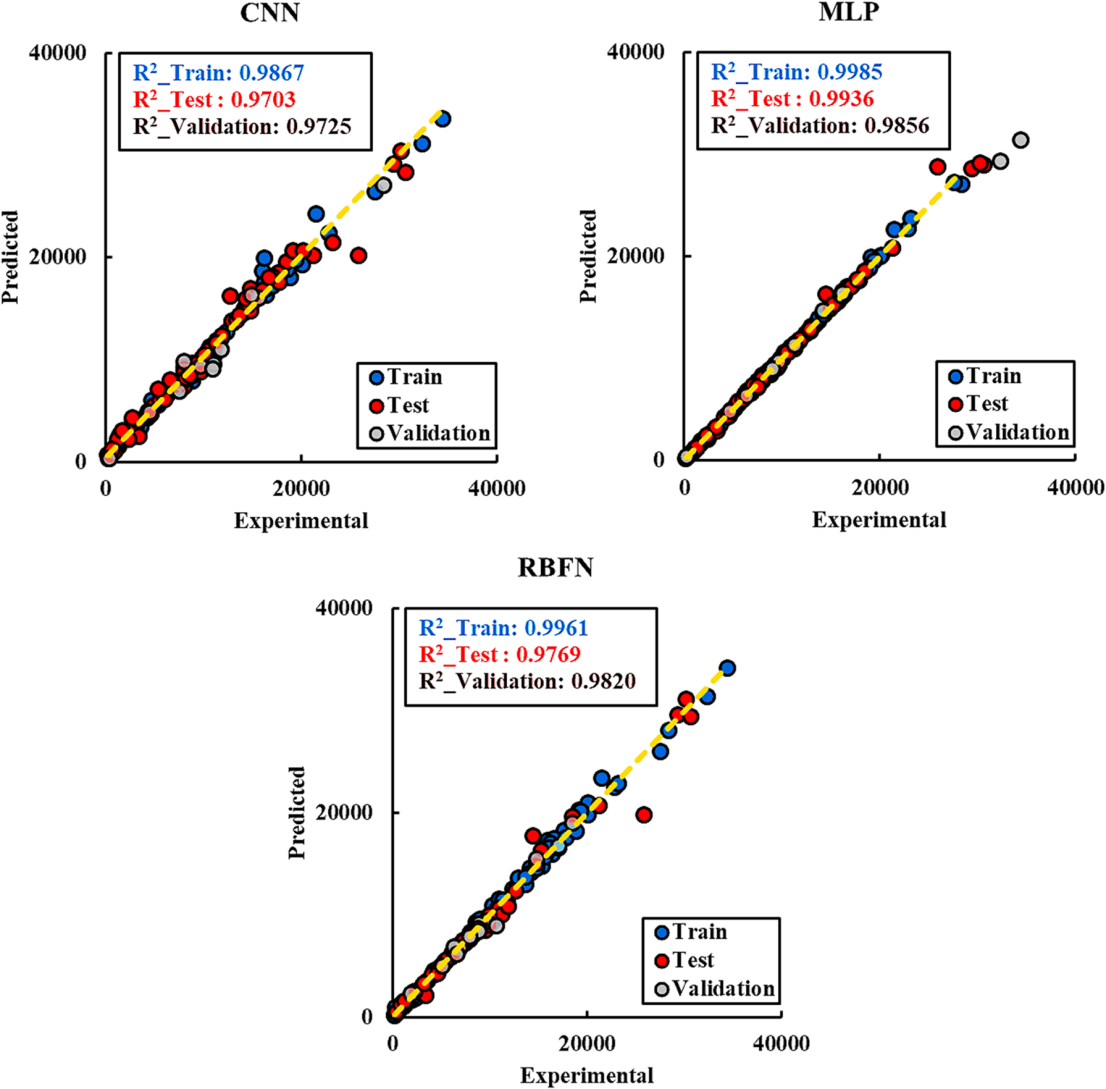
Regression plots comparing predicted and actual QL values for CNN, MLP, and RBFN models in training, validation, and testing phases. The MLP demonstrates the strongest alignment with experimental values (R^2^ ≈ 1), CNN follows closely, while RBFN shows greater scatter, particularly in the testing phase.

Figure 5 presents the regression plots of the CNN, MLP, and RBFN models for the training, validation, and testing datasets. Each plot shows the alignment between predicted and actual liquid production rates (QL), with the R^2^ value quantifying model accuracy. The MLP model exhibits the highest consistency, with R^2^ values close to 1.0 across all three phases, indicating excellent predictive accuracy and strong generalization to unseen data. The CNN model also performs well, particularly during the training and validation phases, but its testing R² values are slightly lower than those of MLP, suggesting minor reductions in generalization capability. By contrast, the RBFN model shows noticeably lower R^2^ values and greater scatter in the regression plots, especially for the testing dataset, reflecting its weaker ability to capture nonlinear relationships in the data. Overall, these results confirm that the GA-optimized MLP delivers the most robust and accurate predictions, followed by CNN, while RBFN lags behind in both accuracy and reliability.

The coefficient of determination, denoted as R^2^, is a widely used metric for measuring the effectiveness of predictive models. It indicates the proportion of variation in the dependent variable that can be explained by the independent variables. A value close to 1 suggests that the model has strong predictive capability, capturing most of the variability in the data. In contrast, an R^2^ value near 0 implies poor model performance, indicating that the model fails to capture the underlying trends, resulting in less accurate predictions.

10 $$\:{R}^{2}=1-\frac{\sum\:_{i=1}^{N}{({y}_{i}^{Pred}-{y}_{i}^{exp})}^{2}}{\sum\:_{i=1}^{N}{({y}_{i}^{Pred}-{average(y}_{i}^{exp}))}^{2}}$$

Residual plots are valuable tools for assessing the reliability and precision of predictive models. They illustrate the differences between the observed (actual) values and the values predicted by the model, enabling the detection of anomalies, biases, or systematic patterns in prediction errors. A well-performing model typically produces residuals that are randomly dispersed around zero, suggesting that it has effectively captured the underlying relationships in the data without introducing systematic error.

**Fig. 6:**
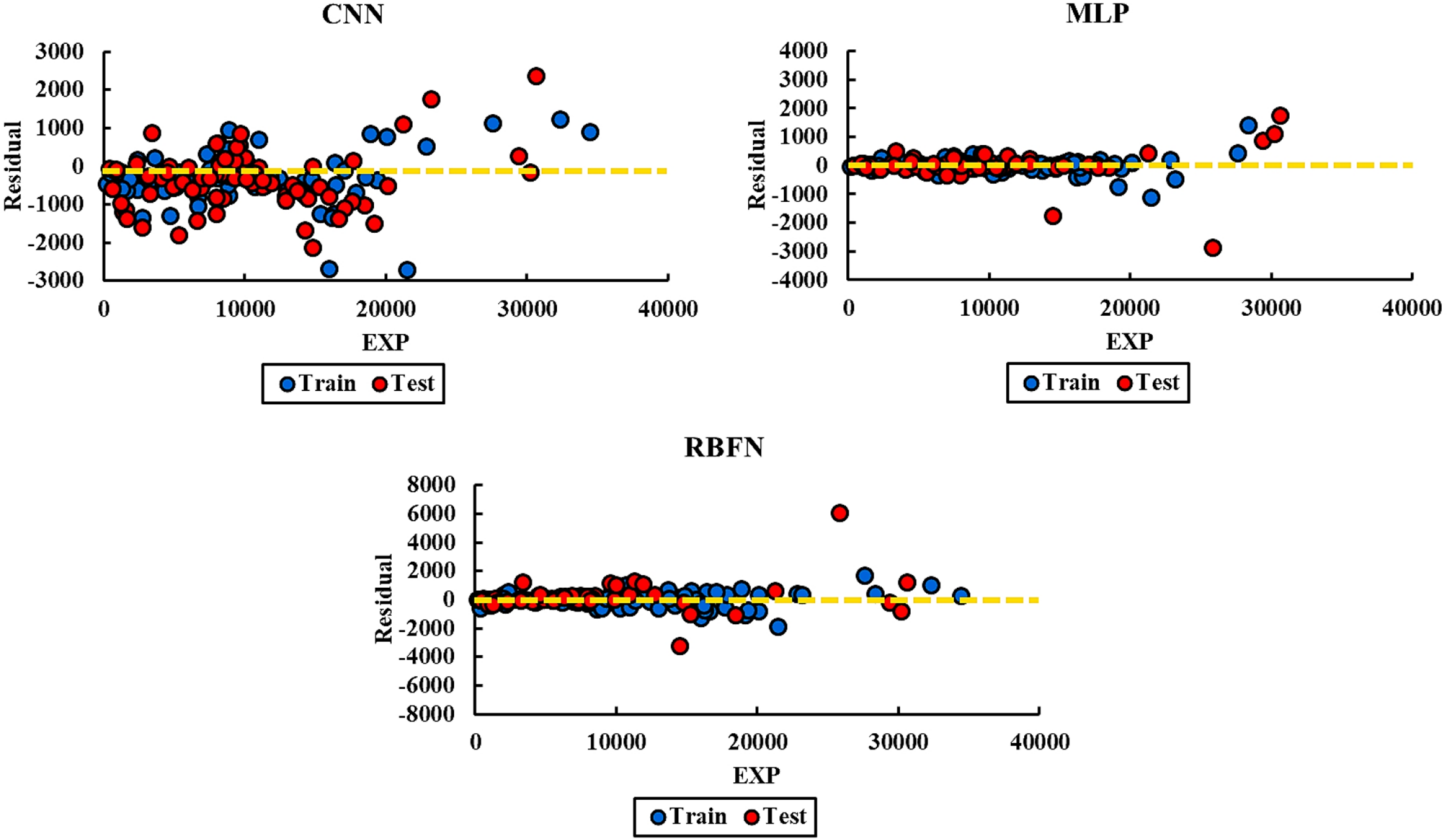
Residual plots of predicted versus experimental QL values for CNN, MLP, and RBFN models in training and testing phases. Residuals of the MLP are narrowly distributed around zero, indicating minimal bias, while CNN shows moderate scatter and RBFN exhibits the widest error spread.

Figure 6 illustrates the residual plots for the CNN, MLP, and RBFN models, comparing predicted liquid production rates (QL) with experimental values across both training and testing datasets. Ideally, residuals should be randomly distributed around zero, indicating unbiased predictions. Among the three models, the MLP shows the narrowest spread, with most residuals concentrated near zero and rarely exceeding ± 1000, highlighting its superior accuracy and stability. The CNN model also demonstrates reasonably well-distributed residuals, though with slightly larger deviations compared to MLP, particularly in the testing phase. By contrast, the RBFN model exhibits a wider scatter, with several points extending close to ± 3000, reflecting weaker predictive capability and higher error variance. Overall, the residual analysis confirms that the GA-optimized MLP provides the most reliable predictions, followed by CNN, while RBFN shows the least consistent performance.

The practical implications of the present findings are directly relevant to petroleum production operations. Accurate prediction of flow rates through choke valves enables engineers to optimize choke settings, thereby reducing the risk of excessive pressure drops that could compromise pipeline integrity and safety. In offshore environments, where choke valves are critical for controlling wellhead conditions under variable flow regimes, reliable forecasting tools help to prevent hydrate formation, mitigate erosion and sand production, and avoid unstable flow cycling. Moreover, machine learning models, such as the GA-optimized MLP developed in this study, can be embedded into digital oilfield systems or real-time monitoring platforms to support proactive decision-making. This integration allows operators to adjust choke configurations dynamically, improving production efficiency while ensuring safer operation of subsea facilities and surface pipelines.

### Statistical criteria in measuring the accuracy of artificial neural network methods

#### Kernel density estimation plots

**Fig. 7:**
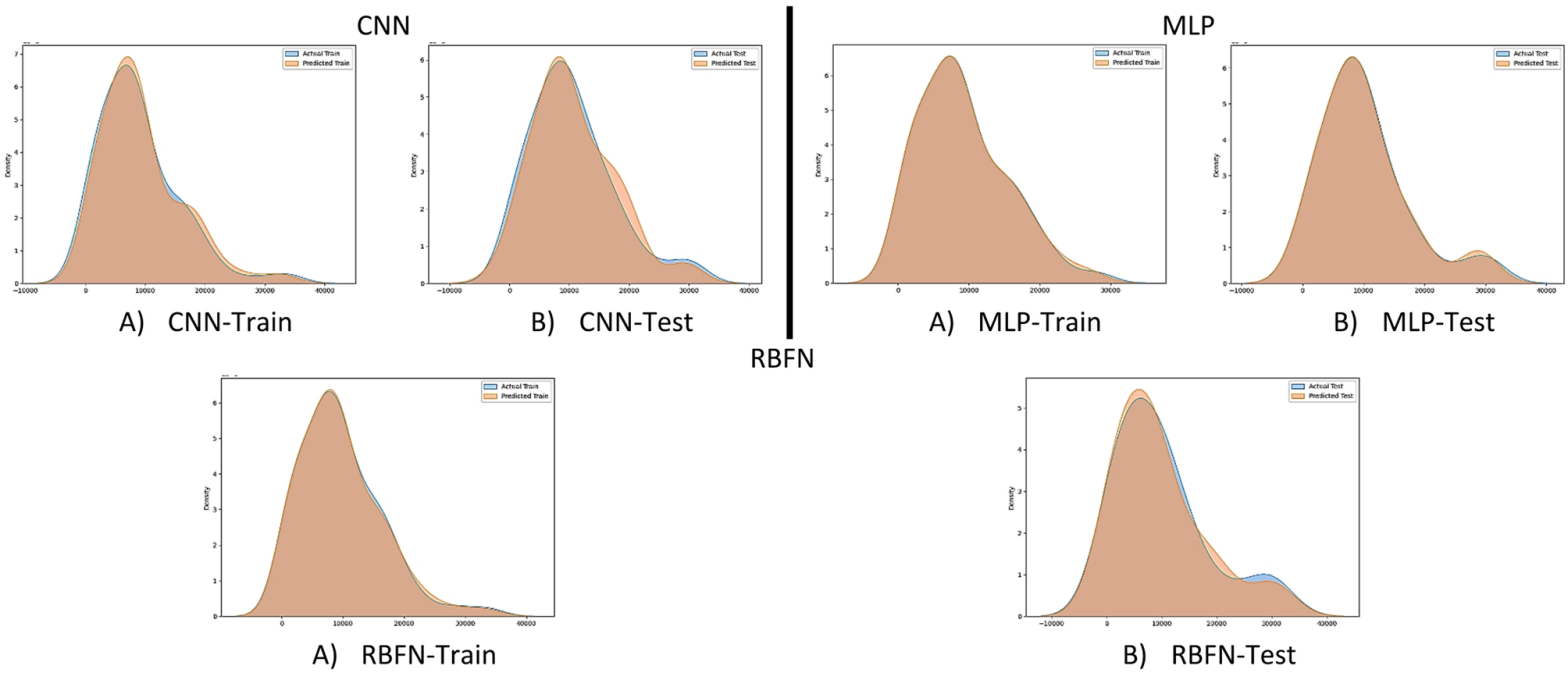
KDE plots comparing predicted and actual QL distributions for CNN, MLP, and RBFN models in training and testing datasets. The MLP curves overlap almost perfectly with actual data, confirming superior accuracy, whereas CNN shows minor deviations and RBFN exhibits noticeable mismatches.

Figure 7 presents the Kernel Density Estimation (KDE) plots comparing the probability distributions of predicted and actual QL values for both training and testing datasets. Ideally, a strong overlap between the predicted and actual curves indicates that the model has accurately captured the statistical distribution of the target variable. Among the three models, the MLP demonstrates the closest alignment, with the predicted and actual distributions nearly overlapping in both training and testing phases, confirming its superior accuracy and generalization. The CNN model also shows a reasonable match between predicted and actual values, though small deviations are visible in the testing dataset, reflecting slightly reduced performance compared to MLP. In contrast, the RBFN model exhibits noticeable differences between the predicted and actual distributions, particularly in the testing phase, indicating weaker predictive reliability. Overall, the KDE analysis reinforces that the MLP model provides the most faithful reproduction of real data patterns, followed by CNN, while RBFN shows the least consistency.

#### Evaluation of statistical error indicators

RMSE (Root Mean Square Error), MAE (Mean Absolute Error), MSE (Mean Squared Error), and MAPE (Mean Absolute Percentage Error) are commonly employed metrics for evaluating the performance of predictive models, especially in regression tasks. These measures are used to assess the extent of deviation between predicted values and actual observations, with each metric offering unique insights into model error. RMSE, in particular, calculates the square root of the average squared differences between predicted and observed values. Due to the squaring of errors before averaging, RMSE places more emphasis on larger errors, making it especially sensitive to outliers and significant discrepancies. The formula for calculating RMSE is as follows:

11 $$\:RMSE=\sqrt{\frac{1}{n}\sum\:_{i=1}^{n}{\left({y}_{i}-{\widehat{y}}_{i}\right)}^{2}}$$

Where $\:{y}_{i}$ represents the actual value, $\:{\widehat{y}}_{i}$ is the predicted value, and $\:n$ is the number of observations.

The Mean Absolute Error (MAE) measures the average magnitude of errors in a set of predictions without considering their direction. It is computed by taking the mean of the absolute differences between predicted and actual values. In contrast to RMSE, MAE treats all errors equally and is less sensitive to large deviations or outliers. The expression used to calculate MAE is as follows:

12 $$\:MAE=\frac{1}{n}\sum\:_{i=1}^{n}\left|{y}_{i}-{\widehat{y}}_{i}\right|$$

This metric is straightforward to understand and provides a linear assessment, where each error has an equal impact on the overall mean.

The Mean Squared Error (MSE) is a commonly used performance metric that calculates the average of the squared differences between predicted and actual values. Like RMSE, it emphasizes larger errors by squaring the deviations, which increases the penalty for significant inaccuracies. However, because the errors are squared, the final result is expressed in squared units, differing from the original data’s unit. The MSE is determined using the following formula:

13 $$\:MSE=\frac{1}{n}\sum\:_{i=1}^{n}{\left({y}_{i}-{\widehat{y}}_{i}\right)}^{2}$$

MSE is often utilized in optimization processes because it is differentiable, a key characteristic required by many machine learning algorithms for effective training.

The Mean Absolute Percentage Error (MAPE) expresses prediction accuracy as a percentage by averaging the absolute percentage differences between actual and predicted values. Its independence from data scale makes it especially useful for evaluating and comparing model performance across different datasets. The formula for MAPE is as follows:

14 $$\:MAPE=\frac{100\%}{n}\sum\:_{i=1}^{n}\left|\frac{{y}_{i}-{\widehat{y}}_{i}}{{y}_{i}}\right|$$

Nevertheless, MAPE has certain drawbacks, particularly when actual values are near zero, as this can lead to extremely large or even undefined percentage error values.

**Fig. 8:**
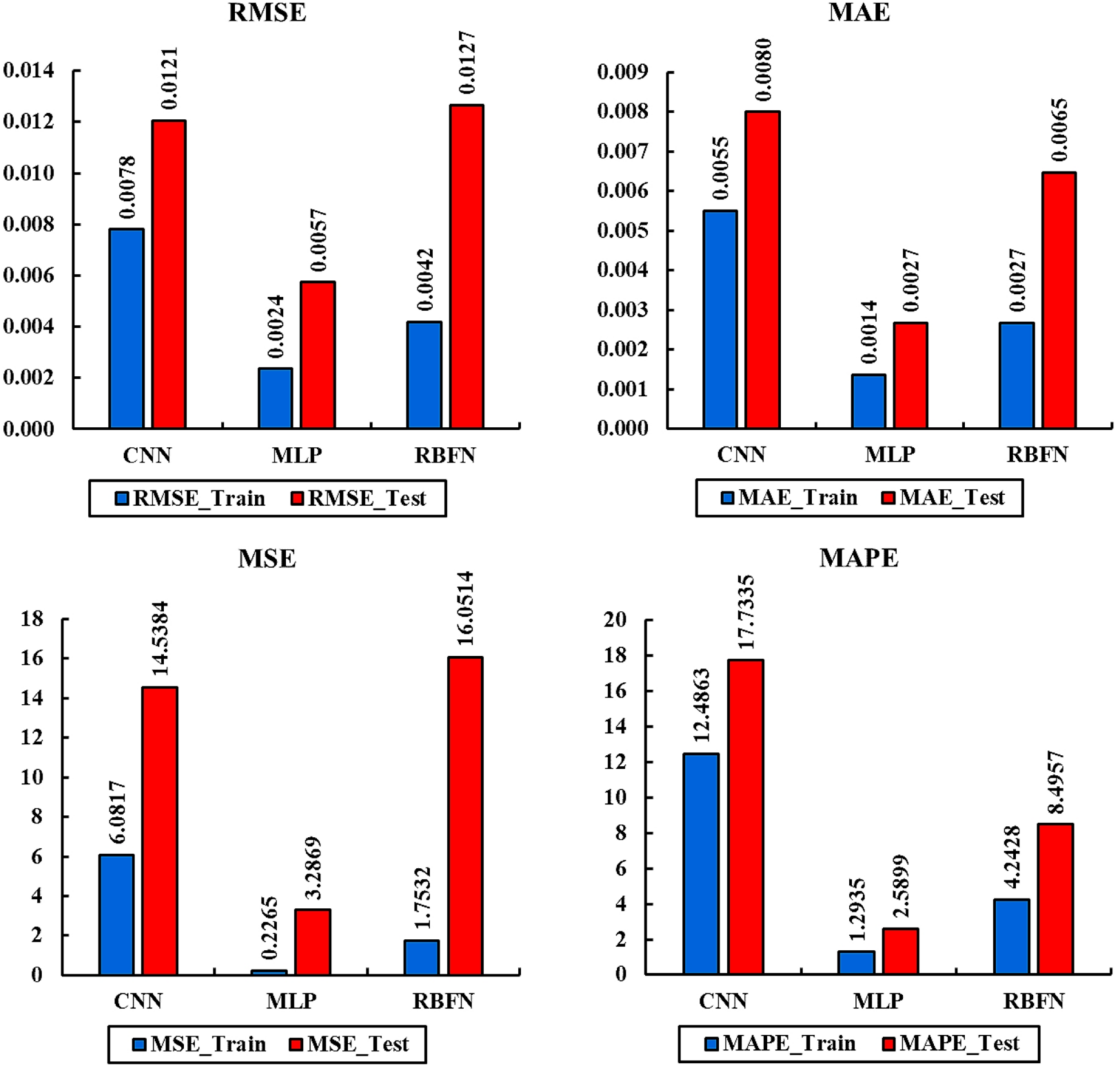
Error metrics (RMSE, MAE, MSE, and MAPE) for CNN, MLP, and RBFN models in both training and testing datasets. The MLP consistently records the lowest error values, CNN maintains intermediate performance, and RBFN produces the largest errors, confirming weaker predictive reliability.

Figure 8 compares the performance of the CNN, MLP, and RBFN models using four standard error metrics—RMSE, MAE, MSE, and MAPE—for both training and testing datasets. A lower value of these metrics indicates higher predictive accuracy and better reliability. Among the three models, the MLP consistently records the lowest error values across almost all metrics, particularly in the testing phase, which highlights its superior ability to generalize to unseen data. The CNN model also shows competitive results, with moderate error values that are lower than those of RBFN but slightly higher than MLP, suggesting good but not optimal performance. By contrast, the RBFN model exhibits noticeably higher error values in both training and testing phases, reflecting reduced predictive capability and less robustness. Overall, the error metric comparison confirms that MLP provides the most accurate and reliable predictions, followed by CNN, while RBFN delivers comparatively weaker performance.

To address measurement quality, the uncertainty and repeatability of the key experimental parameters were evaluated. Table 5 summarizes the typical accuracy ranges of the instruments and test methods used to obtain the dataset, including wellhead pressure, choke size, liquid production rate, gas–liquid ratio, and BS&W content. Pressure measurements were obtained from calibrated wellhead gauges with an uncertainty of approximately ± 0.5% of full scale. Liquid and gas flow rates were determined from test separators with typical uncertainties of ± 2–3%, while GLR was derived accordingly. BS&W content was analyzed in the laboratory using standard procedures, with a repeatability within ± 1 vol%. This error assessment ensures that the dataset used for machine learning remained within acceptable engineering tolerances, supporting the reliability of the developed models.

**Table 5:** Measurement uncertainty and repeatability of key parameters.

Parameter	Measurement method/source	Accuracy (±)	Repeatability
Wellhead pressure (Pwh)	Pressure gauge/sensor at wellhead	± 0.5% of full scale ( ≈ ± 5 bar)	Within ± 2%
Choke size (D64)	Calibrated choke bean size	± 0.1 mm	High (fixed orifice)
Liquid flow rate (QL)	Separator test/calibrated flow meter	± 2% of reading	± 3% across repeated runs
Gas–liquid ratio (GLR)	Calculated from gas and liquid test separators	± 3%	± 3–5%
BS&W content	Standard lab analysis (centrifuge/ASTM method)	± 0.5 vol%	± 1 vol%

### SHAP-based feature importance analysis

SHAP (SHapley Additive exPlanations) is a powerful and model-agnostic interpretability tool rooted in cooperative game theory. It assigns an importance value to each input feature by calculating Shapley values, which represent the average marginal contribution of a feature across all possible feature combinations. This ensures a fair and balanced assessment of each feature’s influence on the model’s output. SHAP is particularly advantageous for interpreting complex and nonlinear machine learning models, where traditional explain ability methods often fall short. It enables both global insights into model behavior and localized explanations for individual predictions. The SHAP summary plot is a valuable visualization that captures the influence, direction, and magnitude of feature effects across the entire dataset.

**Fig. 9:**
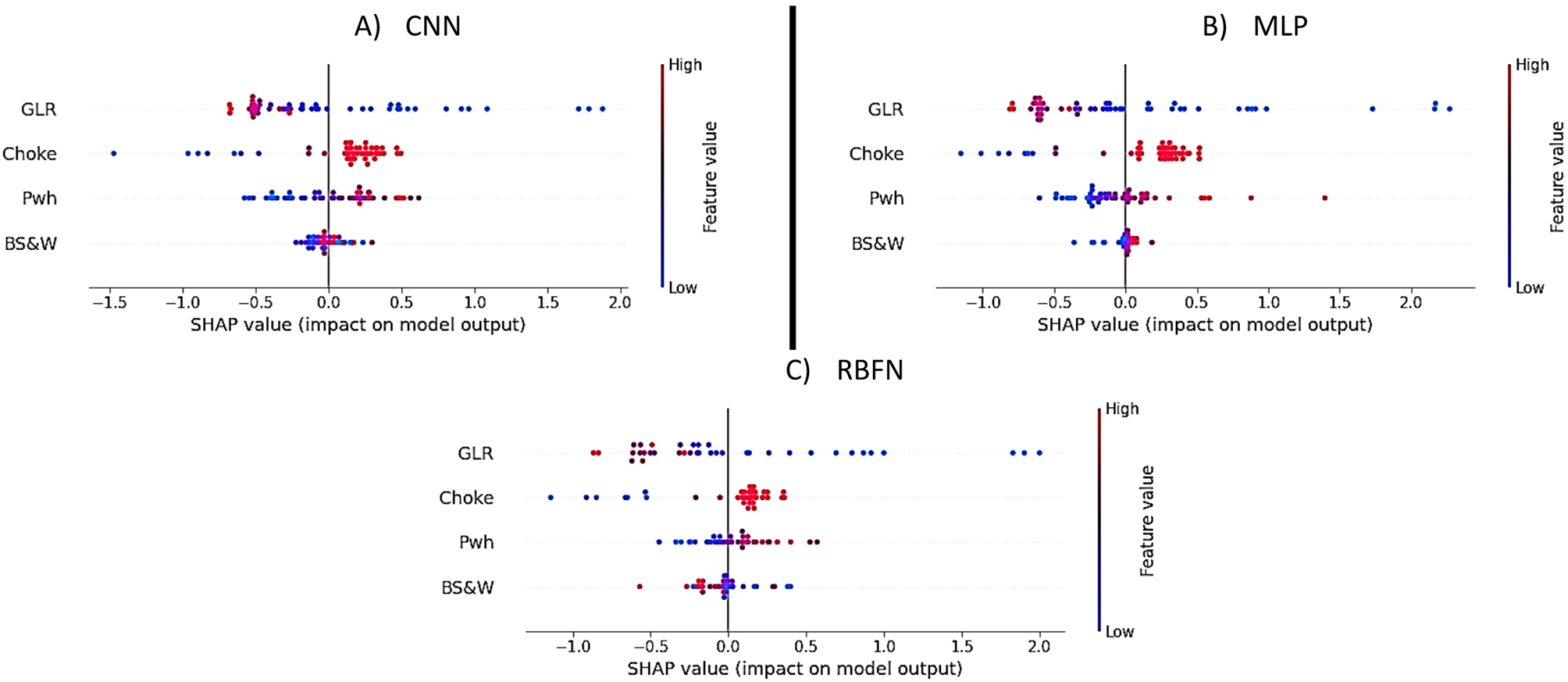
SHAP summary plots for all five models (RBFN, MLP, and CNN), illustrating the contribution of each input feature to the predicted QL. The SHAP values reflect the impact of features on model outputs, with feature values color-coded from low (blue) to high (red).

As illustrated in Fig. 9, SHAP analysis was employed to interpret and contrast the decision-making patterns of three artificial neural network (ANN) models: RBFN, MLP, and CNN. Through SHAP value visualizations, the study assessed how each model weighs the input features when predicting QL. This method offers deeper insights into the dependence of each model on specific inputs and reveals their sensitivity to changes in feature values.

Figure 9A presents the SHAP summary plot for the CNN model, showing the relative importance of input features in predicting liquid production rate (QL). The x-axis represents SHAP values, where positive values increase and negative values decrease the predicted QL, while point colors (red = high, blue = low) indicate feature magnitudes. The results demonstrate that GLR has the strongest negative influence, as higher gas fractions consistently reduce predicted QL, whereas choke size exerts a strong positive impact, with larger openings increasing production. Wellhead pressure (Pwh) shows a moderate and mixed effect, reflecting its indirect role in flow regulation, while BS&W contributes weakly and predominantly negatively, indicating reduced QL at higher water and sediment content. Overall, the SHAP analysis confirms that GLR and choke size are the dominant factors shaping CNN predictions, aligning with the physical mechanisms of choke flow behavior.

Figure 9B shows the SHAP summary plot for the MLP model, illustrating the relative impact of each input feature on the prediction of liquid production rate (QL). The x-axis represents SHAP values, where positive values increase and negative values decrease the predicted QL, while the point colors indicate feature magnitudes. The results indicate that choke size is the most influential factor, with larger choke openings (red points) strongly associated with higher QL predictions. GLR also plays a significant role but with a negative effect, as higher gas fractions lead to reduced QL, consistent with multiphase flow dynamics. Wellhead pressure (Pwh) demonstrates a moderate but positive contribution, particularly at higher values, suggesting its role in stabilizing and enhancing flow. BS&W exhibits a relatively minor influence, generally associated with negative SHAP values, meaning increased water and sediment content lowers QL. Overall, the SHAP interpretation confirms that the MLP model relies primarily on choke size and GLR, with Pwh providing additional support, which aligns with field observations of choke-controlled production behavior.

Figure 9C illustrates the SHAP summary plot for the RBFN model, highlighting the contribution of each input feature to predicting the liquid production rate (QL). The x-axis shows SHAP values, where positive values increase the predicted QL and negative values decrease it, while the colors represent feature magnitudes. The results reveal that GLR exerts a strong negative influence, with higher gas fractions consistently reducing predicted QL. Choke size appears as the next most important feature, with larger openings generally contributing to higher QL, although the effect is less pronounced compared to the MLP and CNN models. Wellhead pressure (Pwh) shows only a modest and scattered influence around zero, indicating limited predictive importance within the RBFN framework. Similarly, BS&W demonstrates a relatively minor negative effect, with higher water and sediment content lowering QL. Overall, the SHAP analysis confirms that while RBFN identifies GLR and choke size as key factors, its weaker differentiation among the secondary variables (Pwh and BS&W) explains the comparatively lower accuracy of this model relative to CNN and MLP.

A sensitivity discussion based on the SHAP analysis was also conducted to identify the most influential input parameters on choke flow behavior. The results indicate that choke size (geometry) and wellhead pressure are the dominant factors controlling liquid production rate, as they directly determine the available flow area and driving pressure differential. The gas fraction (GLR) also exerts a strong influence by modifying flow regime transitions and affecting slip between phases, particularly at higher values where earlier choking onset is observed. In comparison, BS&W content showed only a secondary impact within the studied range. Overall, the sensitivity results confirm that geometric and pressure-related parameters govern the primary choking behavior, while fluid composition (gas fraction) plays a key role in determining the stability of multiphase flow through the choke.

To enhance the generalizability of the predictive models across different operational scales, the dimensional input parameters were converted into a dimensionless framework. The effective choke diameter $\:D$ was selected as the characteristic length, and the average superficial velocity was calculated as:

15 $$\:A=\frac{\pi\:{D}^{2}}{4}$$

16 $$\:v=\frac{{Q}_{L}+{Q}_{G}}{A}$$

Based on these definitions, the Reynolds, Froude, and Weber numbers were computed as follows:

17 $$\:Re=\frac{{\rho\:}_{m}vD}{{\mu\:}_{m}}$$

18 $$\:Fr=\frac{v}{\sqrt{gD}}$$

19 $$\:We=\frac{{\rho\:}_{m}{v}^{2}D}{\sigma\:}$$

Here, $\:{\rho\:}_{m}$ and $\:{\mu\:}_{m}$ denote the mixture density and viscosity, estimated using volume-fraction-weighted averages, and $\:{Q}_{G}$ was derived from the gas–liquid ratio (GLR) after appropriate unit conversion. In addition to predicting the dimensional liquid production rate ($\:{Q}_{L}$), the dimensionless discharge coefficient was also evaluated as a normalized target variable:

20 $$\:{C}_{d}=\frac{Q}{A\sqrt{2\varDelta\:P/{\rho\:}_{m}}}$$

Incorporating these dimensionless parameters into the model inputs enhanced the scalability of the results and facilitated meaningful comparisons between laboratory-scale and field-scale choke flow conditions.

The ratios of gas-to-liquid density and viscosity play a critical role in determining choke flow behavior. A higher gas–liquid density contrast generally promotes slip between phases, intensifying phase segregation and altering the pressure drop characteristics across the choke. Conversely, when gas and liquid densities are closer, the flow tends to be more homogeneous, and the empirical correlations derived for one fluid system may not directly transfer to another. Similarly, viscosity ratio influences the momentum transfer between phases: low-viscosity gases flowing with high-viscosity liquids often lead to enhanced turbulence and interfacial shear, while systems with closer viscosity values exhibit more stable flow patterns. Although the present dataset is specific to the studied reservoir fluids, the dimensionless framework (e.g., Reynolds, Froude, Weber numbers) provides a pathway for extending these results to other fluid pairs. By expressing the governing parameters in terms of density and viscosity ratios, the predictive models can be scaled to different hydrocarbon–gas or oil–water systems with appropriate adjustment.

To benchmark the proposed machine learning models, a comparison was made with representative empirical correlations commonly applied in choke flow prediction, such as those reported by Gilbert and Mirzaei-Paiaman et al. These classical correlations provide a quick estimate of flow rate based on choke size, pressure, and fluid properties, but they typically assume simplified flow regimes and may not fully capture nonlinear interactions among parameters. In contrast, the machine learning models developed in this study, particularly the MLP architecture, demonstrated significantly higher predictive accuracy with lower RMSE and higher R^2^ values. This comparison highlights that while empirical models remain useful for preliminary design and quick screening, data-driven approaches offer improved robustness and reliability when dealing with complex multiphase flow conditions.

## Conclusions

And with the epoch of increasing energy needs on the grounds of smart recovery of hydrocarbon reservoirs, the ability to foresee accurately the wellhead production rate via chokes is the basis of reservoir management and recoverability optimization. It’s not an improvement it’s the secret to optimization of operating costs, recovery maximization of the resource, and surface facility safety. Through facilitating management of higher-order multiphase flow regimes, chokes avert water and gas coning loss of sand production and hence guarantee long-term asset integrity. The new approach used in this study using the combination of newer neural networks and genetic algorithm optimization, apart from providing additional diagnosis and enhanced predictions, also outperformed traditional empirical models. It is revolutionary smart reservoir management model, or sustainable fact-based decision-making and production. Most especially and rightly, the system prolongs reservoir lives and adds new value to an oil industry digital revolution in which each drop of precision sets the course of the energy future.

This study proposes an innovative framework for predicting the liquid production rate (QL) using three popular ANN models: MLP, RBFN, and CNN. A distinguishing feature of this research is the use of GA for hyperparameter tuning across all three models, enabling optimal configuration and improved predictive performance. The input variables used in the modeling include wellhead pressure (Pwh), choke size (D64), basic sediment and water content (BS&W), and gas–liquid ratio (GLR). Each parameter comprises 182 data points, yielding a total of 910 samples. The dataset was divided into training, validation, and testing subsets to ensure reliable and generalizable model assessment.

Model performance was evaluated using four standard error metrics: MSE, RMSE, MAE, and MAPE. To support the reliability and interpretability of the predictive models, various visualization techniques were employed, including KDE plots, learning curves, regression plots, error distribution diagrams, and SHAP value analysis. These tools not only helped to validate model performance but also provided valuable insights into the influence and contribution of each input variable at both global and individual levels.

Among the tested models, the MLP architecture demonstrated superior performance. It achieved R2 values of 0.9985 for the training set, 0.9856 for the validation set, and 0.9936 for the testing set. The associated error metrics further confirmed the accuracy of the MLP model, with RMSE values of 0.0024 (training) and 0.0057 (testing), MAE values of 0.0014 (training) and 0.0027 (testing), MSE values of 0.2265 (training) and 3.2869 (testing), and MAPE values of 1.2935% (training) and 2.5899% (testing). These outcomes highlight the robustness and high predictive capability of the GA-optimized MLP model in forecasting liquid production rate based on operational well parameters.

Even though the results are encouraging, the work is incomplete and limited. The Reshadat oil reservoir database and 182 test records from seven wells cannot be representative of all the diversity of world reservoir conditions and therefore cannot be extended to other fields with other fluid properties or other choke types. GA hyperparameter tuning, while as useful as it is, is computationally expensive and needs to be carefully tuned so that the solution does not degenerate or converge prematurely. The models also rely on main or subcritical flow conditions without live-parameter variation for parameters such as temperature or emulsion concentration and can yield prediction bias in dynamic operating conditions. Interpretability does not pose an issue here since SHAP analysis is insightful albeit sparsely exhaustive exposing the “black-box” status of ANNs, and dependence on normalization in preprocessing can be used to make errors more if data quality problems such as missing values or outliers are not solved.

This process can be extended with broader and more representative samples from other regions of the world to reduce even more detailed and broad models further. Additional input parameters such as temperature, API gravity, or live sensors will show increasingly broader predictability under differing conditions. Hybrid model procedures combining both ANNs and physics simulation will prevent loss of interpretability at the expense of high accuracy. In addition, integration of these next-gen models with real-time monitoring using edge computing or API integrations will also help in the optimization of production. Finally, comparison of next-gen methods like transformer networks or ensemble methods can even identify even better approaches for multiphase flow prediction under poor-quality reservoir conditions.

In future work, the present findings could be validated and extended using advanced computational and data-driven approaches. High-fidelity CFD simulations can provide detailed insights into flow structures, pressure distribution, and regime transitions inside choke valves, serving as a complementary tool to verify the predictive accuracy of machine learning models. At the same time, more advanced ML architectures such as ensemble learning or transformer-based networks may further enhance generalization across different reservoirs and operating conditions. Integrating these methods with the proposed framework will strengthen confidence in the results and facilitate broader application of predictive models for choke flow optimization.

## Data Availability

The datasets used and/or analyzed during the current study are cited and referenced in this article.

## Abbreviations

ANN: Artificial Neural Network
BS&W: Basic Sediment and Water Content
CNN: Convolutional Neural Network
GA: Genetic Algorithm
GLR: Gas–Liquid Ratio
KDE: Kernel Density Estimation
MAE: Mean Absolute Error
MAPE: Mean Absolute Percentage Error
MLP: Multilayer Perceptron
MSE: Mean Square Error
Pwh: Wellhead Pressure
SCADA: Supervisory Control and Data Acquisition
RBFN: Radial Basis Function Network
SHAP: SHapley Additive exPlanations
